# Copper-assisted oxidation of catechols into quinone derivatives†

**DOI:** 10.1039/d0sc04883f

**Published:** 2020-12-21

**Authors:** Ana Cristina Gómez-Herrero, Carlos Sánchez-Sánchez, Frédéric Chérioux, Jose Ignacio Martínez, José Abad, Luca Floreano, Alberto Verdini, Albano Cossaro, Estelle Mazaleyrat, Valérie Guisset, Philippe David, Simone Lisi, José Angel Martín Gago, Johann Coraux

**Affiliations:** Univ. Grenoble Alpes, CNRS, Grenoble INP, Institut NEEL 38000 Grenoble France johann.coraux@neel.cnrs.fr; Materials Science Factory, Instituto de Ciencia de Materiales de Madrid-CSIC C/Sor Juana Inés de la Cruz 3 Madrid 28049 Spain; Univ. Bourgogne Franche-Comté, FEMTO-ST, CNRS, UFC 15B avenue des Montboucons F-25030 Besançon Cedex France; Departamento de F, ica Aplicada, Universidad Politécnica de Cartagena Calle Doctor Fleming, s/n 30202 Cartagena Spain; Laboratorio TASC, CNR-IOM Basovizza SS-14, Km 163.5 34149 Trieste Italy; Univ. Grenoble Alpes, CEA, IRIG/DEPHY/PHELIQS 38000 Grenoble France; Institute of Physics, Academy of Sciences of the Czech Republic 162 00 Praha Czech Republic

## Abstract

Catechols are ubiquitous substances often acting as antioxidants, thus of importance in a variety of biological processes. The Fenton and Haber–Weiss processes are thought to transform these molecules into aggressive reactive oxygen species (ROS), a source of oxidative stress and possibly inducing degenerative diseases. Here, using model conditions (ultrahigh vacuum and single crystals), we unveil another process capable of converting catechols into ROSs, namely an intramolecular redox reaction catalysed by a Cu surface. We focus on a tri-catechol, the hexahydroxytriphenylene molecule, and show that this antioxidant is thereby transformed into a semiquinone, as an intermediate product, and then into an even stronger oxidant, a quinone, as final product. We argue that the transformations occur *via* two intramolecular redox reactions: since the Cu surface cannot oxidise the molecules, the starting catechol and the semiquinone forms each are, at the same time, self-oxidised and self-reduced. Thanks to these reactions, the quinone and semiquinone are able to interact with the substrate by readily accepting electrons donated by the substrate. Our combined experimental surface science and *ab initio* analysis highlights the key role played by metal nanoparticles in the development of degenerative diseases.

## Abstract 2

An antioxidant catechol transforms following intramolecular redox reactions into highly reactive oxygen species, a semiquinone and a quinone, on copper.

## Graphical abstract

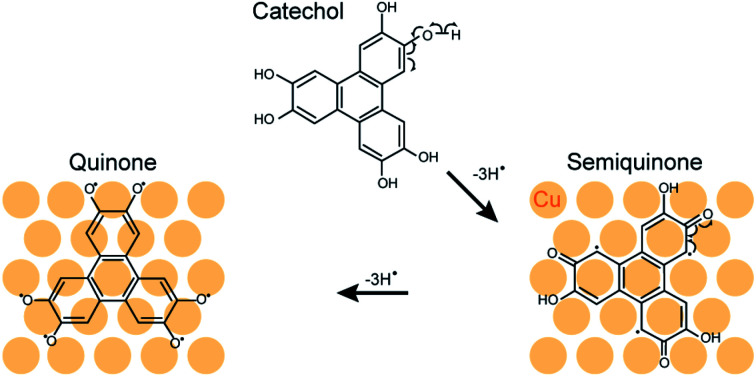

## Introduction

Benzenediols, including their *ortho* isomers, catechols, and their derivatives such as flavonoids^1^ and estrogens,^2^ are antioxidants capable of counteracting the deleterious role played by free radicals in degenerative diseases. Oxidation of one or two of the alcohol functions of a catechol, *via* dehydrogenation reactions, yields a semiquinone and a quinone, respectively.^3^ Both are strong oxidants, so-called reactive oxygen species (ROSs), altering the structure of DNA and being usual suspects in the development of cancers.^4–10^ Inside living organisms, even partial conversion of catechols into (semi)quinones is liable to produce an oxidative stress, possibly relevant in a variety of diseases, not limited to cancers.

The production of ROSs corresponds to an exchange of electrons, an oxidation of a species (loss of electrons) yielding the ROS together with a reduction (gain of electrons) of another species. Inside a biological cell, the presence of an external agent (chemical compound, nanoparticle, biological species, *etc.*) can activate a reduction channel, thereby triggering the concomitant formation of ROSs *via* oxidation. Such processes are usually described by Fenton^11^ and Haber–Weiss^12^ reactions (see ESI, Fig. S1 and S2†). In the case of the catechol-to-(semi)quinone conversion, the redox reactions involve metal cations^5,13,14^ as reducing agents. Such reactions may occur with metal cations in solution or at the surface of metal oxide nanoparticles.^15,16^ Surprisingly, metallic (*i.e.* non ionic) nanoparticles are more cytotoxic than their corresponding metal cations in solution.^17^ Some explanations have been proposed, including size effects, specific physical or chemical properties of their surface, high specific areas, but for ROS production to occur, a reducing agent is needed and the (non ionic) metallic nanoparticles cannot assume this role (they cannot be reduced).^18^ Fenton or Haber–Weiss reactions hence seem irrelevant in this case, and the catalysis of ROS formation with metal nanoparticles and related health issues^18–21^ remain open questions.

**Scheme 1:**
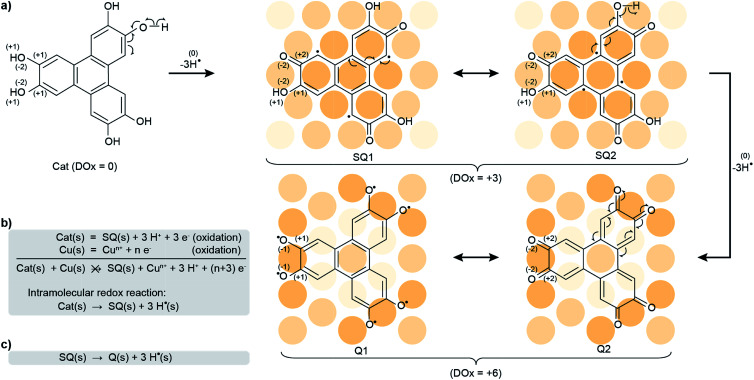
Oxidation of a catechol into a semiquinone and a quinone. (a) Two-step oxidation (with departure of three hydrogen radicals, -3H˙, at each step) of the HHTP molecule, a catechol (Cat), into a semiquinone, of which two mesomers are shown (SQ1, SQ2), and a quinone, of which two mesomers are shown (Q1, Q2). The mesomers each correspond to a distinct charge distribution. Only one third of the charge reorganisation processes are shown. The surface Cu atoms are sketched with orange balls, with the darkest orange shades used to highlight those atoms forming stronger bonds with the molecules. The global degree of oxidation (DOx) is indicated for each molecule, and the individual DOxs of relevant H atoms and one third of the C atoms linked to O atoms, and of these O atoms, are specified in parenthesis. (b) The simultaneous oxidation of both the catechol and the Cu metal would yield the semiquinone and Cu ions, an impossible process. Instead, an intramolecular redox reaction transforms the HHTP molecule into the semiquinone molecule. (c) A similar kind of reaction transforms the semiquinone into the quinone molecule.

At this stage, understanding the fine mechanism *in vivo* and answering this question are hindered by the expectedly complex evolution of the system, following possibly intertwined reaction steps driven by a variety of parameters. Therefore, we have investigated the reorganisation of electron distribution (so-called redox process) of a model system constituted only by a crystalline metal surface (Cu(111)) and a tri-catechol molecule (2,3,6,7,10,11-hexahydroxytriphenylene, HHTP, see Scheme 1a) under ultrahigh vacuum (UHV). We have analysed the chemical transformations *via* a detailed physicochemical study exploiting surface science tools, with emphasis on electron transfers within the molecules and in interaction with their substrate. To characterise these transfers, which govern the chemical transformations, we have highlighted the global and local degree of oxidation (DOx). Using *in situ* synchrotron X-ray photoemission spectroscopy (XPS), near-edge X-ray absorption fine structure (NEXAFS), scanning tunneling microscopy (STM), and density functional theory (DFT) calculations (details are provided in the Experimental and numerical methods section), we establish that, being in its reduced form (catechol), the HHTP molecule can only transform on the surface *via* two successive intramolecular redox reactions, yielding hydrogen radicals (subsequently transformed into dihydrogen) together with the semiquinone and the quinone molecular forms. In this process, which may be regarded as a surface-assisted dehydrogenation,^22–24^ the global DOx of the molecule increases (Scheme 1b and c). The first intramolecular redox reaction (*i.e.* surface-assisted dehydrogenation) occurs spontaneously at room temperature, while the second is already complete after annealing to 530 K. The global DOx of the semiquinone and quinone is slightly reduced compared to the nominal +3 and +6 values (corresponding to 3 or 6 surface-assisted dehydrogenations respectively) due to a counteraction process, whereby the Cu substrate donates about 1 and 2 electrons per molecule, respectively. Our results support that the surfaces of metallic nanoparticles may promote a self-conversion of antioxidant molecules into highly reactive oxygen species, which can explain the role of these types of nanoparticles in the development of degenerative diseases.

## Results and discussion

### On-surface molecular organisation

**Fig. 1:**
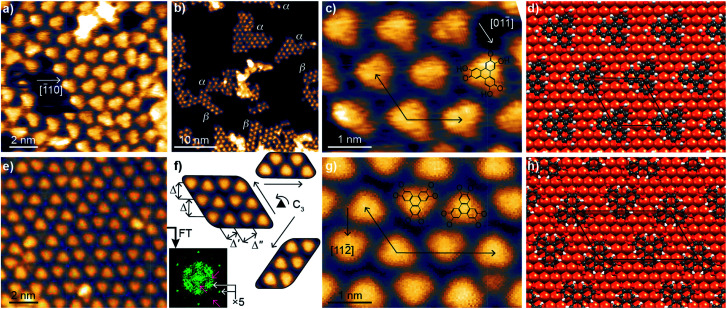
Molecular self-assembly on Cu(111). (a–c) STM images measured at 80 K after room temperature deposition, and (d) optimised ball-model structure of the α-type molecular network obtained from DFT calculations. In (b), islands of the α-type and β-type molecular networks, each with specific crystallographic edges, are visible. (e–g) STM images measured at 80 K after annealing to 530 K, and (h) corresponding optimised ball-model structure obtained from DFT calculations. (f) Fourier transform corresponding to (e) is shown, with white arrows highlighting the ×5 periodicity of the molecular lattice and pink arrows highlighting the less-well-defined longer-range periodicity; STM close-ups on the three equivalent orientations of the molecular self-assembly. For one of these orientations (left), two rows of ziplock-arranged molecules are visible, and characteristic distances (*Δ* = 1.25 nm, *Δ*′ = 0.81 nm, *Δ*′′ = 1.04 nm) are highlighted. In the zooms (c, g), sketches of the structure of individual molecules (semiquinone and quinone, respectively) are overlaid on the STM images, together with a substrate crystallographic direction along a mirror plane of the molecules, and the molecular/substrate superstructure identified with its unit cell vectors. The black rhombus/parallelogram in (d and h) highlight a unit cell of the molecular assembly.

A sub-monolayer coverage of HHTP molecules was evaporated onto a Cu(111) surface held at room temperature (see details in the Experimental section). It was characterised by STM, XPS and NEXAFS. The STM images in Fig. 1a–c reveal the formation of a self-assembled monolayer, with molecules appearing as triangular units. A detailed inspection of the images reveals that they group into domains of size not larger than 10 nm or even less, exhibiting different structural lattices with well-defined crystallographic orientations (α-type and β-type) with respect to the Cu(111) surface. Low-energy electron diffraction patterns show no specific signature of these superstructures, but a strong diffuse scattering background. This absence of signal could be due to the small size of the molecular domains and a lack of structural coherence within these domains (Fig. 1b). After careful correction for thermal drift effects in STM images, we find that the unit cell vectors form a 117 ± 4° angle and have equal length (within the few percent precision of our measurement). This length (∽1.25 nm) is compatible with a (5 × 5) superstructure with respect to the Cu(111) surface (lattice parameter, 0.254 nm). In one of the lattices (α-type), one median of the triangular units matches the [1̄10] crystallographic direction of the substrate, as can be seen on the ball-and-stick model of Fig. 1d. As shown in Fig. S4 (see ESI†), the lowest-energy binding configuration corresponds to a molecule with the center of its central C ring sitting directly atop a Cu atom (Fig. S5† compares this configuration with a higher-energy one, mixing molecules of different orientations). In the second lattice (β-type), the [112̄] direction of the substrate almost matches the orientation of one median of the triangular units (see ESI, Fig. S8†).

After annealing at 530 K, the organisation of the molecules changed (Fig. 1e–h), presenting similarities with those reported for fully-hydrogenated HHTP molecules (catechol form) onto Ag(111) (ref. 25) and Au(111).^26^ Molecular symmetry and organisation hence seem correlated. STM reveals that the motif of the molecular lattice presents a different symmetry than before annealing: the molecular self-assembly assumes three equivalent crystallographic orientations (according to the surface's three-fold symmetry), with two teeth-facing jigsaw-like molecular rows and three characteristic length-scales (Fig. 1f). Correction of thermal drift effects allows us locally identifying unit cell vectors with a length ratio of 1.79 ± 0.08 and forming an angle of 117 ± 4°. These values suggest that (i) the unit vectors align those of the Cu(111) surface (117° ≈ 120°), and (ii) given that the shortest unit vector has a length of ∽1.25 nm five times longer than the unit cell vectors of Cu(111), the longest unit vector is nine times larger than the unit cell vector of Cu(111) (1.79 ≈ 1.8 = 9/5). The unit cell is hence a (5 × 9) for the example shown in Fig. 1f. Our DFT calculations confirm the stability of this structure, represented in Fig. 1h, and the STM image simulations provide a good match with the experiments (compare ESI, Fig. S6c and d to 1f). In both the experiments and simulations, the two kinds of molecules (pointing along or opposite to a 〈112̄〉 direction of Cu(111)) present the same appearance in STM images, consistent with their similar binding to Cu(111). Although the crystallographic orientation of the molecular domains is well-defined, there are local variations of the intermolecular distance, especially along the long period of the lattice, of about 10%. For this reason we also performed DFT calculations for a (5 × 10) structure and simulated the corresponding STM images. The (5 × 10) structure is found stable too, and the image simulations visually also agree well with the STM observations (compare ESI, Fig. S6e and f† to 1f). The molecule's adsorption energies calculated with DFT are similar for molecules in the (5 × 9) and (5 × 10) unit cells, which gives an explanation for the observed structural variability. This variability (×9 *versus* ×10) provides a natural explanation why in reflection high-energy electron diffraction (RHEED) only, a ×5 superstructure is detected (see ESI, Fig. S9†): within the electron beams' coherence length (few 10 nm), the long period of the unit cell varies at the few nanometer scale, in other words the constructive interference conditions cannot be met. The short period (×5), on the contrary, is well-defined and yields a strong diffraction signal. Consistently, the Fourier transform of the STM images (Fig. 1f) shows strong signals stemming from the ×5 periodicity (gray arrows), and much weaker signals from longer-range (presumably ×9 and ×10, pink arrows) periodicities.

### Physicochemical evidences of the oxidation of the molecules

The observed structural modifications of the self-assembly point towards changes in the nature of the molecules upon annealing.^27–29^ These changes may not be directly evident in the molecular orbitals imaged with STM, since these orbitals often spread beyond the atomic scale where chemical modifications occur (our simulations of STM images based on the structural model proposed below confirm this point, see ESI, Fig. S5 and S6†). There is an exception though, when the changes break the initial symmetry of the molecule. In the case of the HHTP molecule, a transformation of one or two of its three “legs” would result in a non-planar binding to the substrate. STM image simulations, performed for related molecule, a tetrahydroxybenzene molecule dehydrogenated in a non-symmetric way on Cu(111), indicate that the apparent height of the molecule should be non-uniform, lower where the hydroxy groups are dehydrogenated and the molecule is more strongly pinned to the surface.^30^ We expect the same kind of effect if the hydroxy groups were dehydrogenated pairwise exclusively at one or two legs of the HHTP molecule, but such a symmetry breaking is not observed, suggesting that the three molecular legs present the same structure through the whole transformation process. To understand the precise nature of the molecular changes and whether/how the DOx of the molecules evolves upon thermal annealing, we turn to a spectroscopic characterisation providing elemental-selective information.

Our XPS measurements reveal qualitatively similar C 1s spectra before/after annealing to 530 K, which seem to comprise (at least) three components, ascribed to C atoms bond to O-containing groups (285.7/285.5 eV), to H atoms in the three outer rings (284.7/284.4 eV), and to C atoms in the central ring (284.2/284.0 eV).^30–33^ In fact, the highest-energy component, corresponding to chemically inequivalent C atoms bound to alcohol or ketone groups, comprise two contributions, which are typically separated by only 0.2 eV from one another (see ESI, Fig. S10†),^34,35^ usually being grouped into a single component.^30–33^

**Fig. 2:**
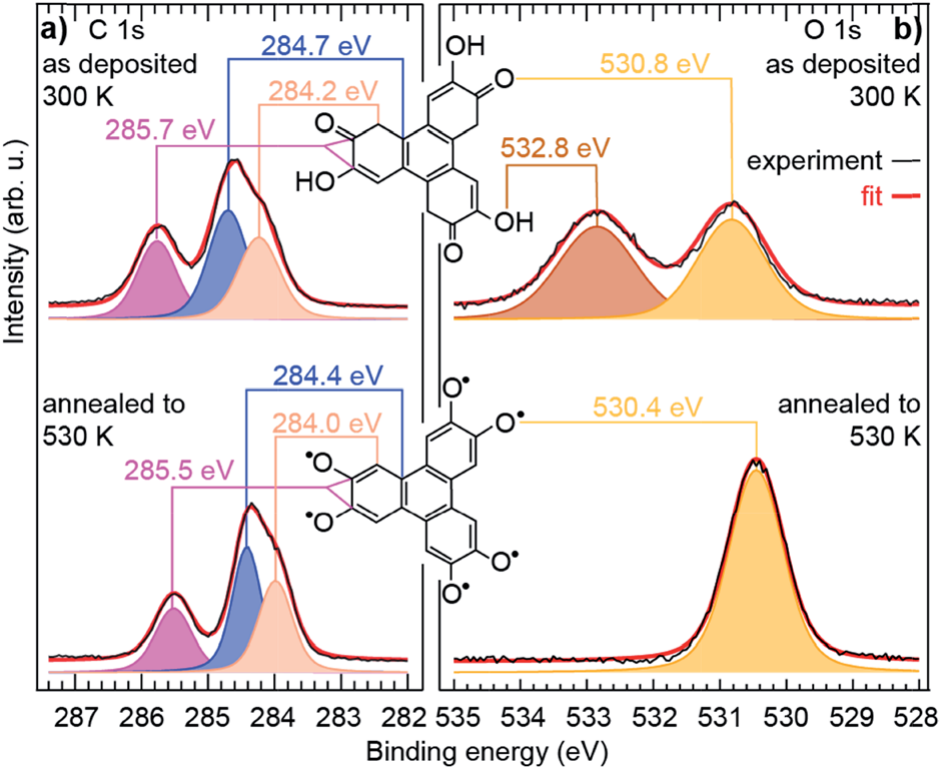
Chemical transformations of the molecule. High resolution (a) C 1s and (b) O 1s core level spectra of a sub-monolayer molecular deposit on Cu(111), before and after annealing to 530 K. The experimental data (black curves) are fit (red curve, see ESI† for details) with several components, each corresponding to chemically inequivalent C or O atoms.

In contrast, profound changes are found in the O 1s spectra (Fig. 2). This clearly indicates that the main thermally-induced chemical modifications of the HHTP molecules involve the alcohol functional groups. The details of the fit analysis of the XPS spectra are given in the ESI, Table S1.† In fact, already at 300 K, the O 1s spectrum comprises two components (532.8 and 530.8 eV) of very similar weight. Two-component spectra of this kind have been assigned to the coexistence of an alcohol function (532–533 eV) and a ketone function (∼531 eV) in related molecules deposited on various metal surfaces.^30–32,36–39^ NEXAFS spectra display signature of an electronic transition towards a C=C–C=O orbital^40^ (see ESI, Fig. S13†), which supports this interpretation. Our analysis altogether suggests that, at 300 K, half the alcohol functions are dehydrogenated and pairs of alcohol and ketones are stabilised, *i.e.* a semiquinone is formed (Scheme 1a).

This semiquinone is oxidised compared to the starting HHTP molecule. Copper (the surface), being in a metal form is not amenable to further reduction, hence the formation of the semiquinone cannot be accounted for by a Cu/HHTP redox reaction, that is thermodynamically irrelevant. We suggest that another kind of redox reaction, an intramolecular redox reaction of the catechol (HHTP) form, which is oxidised (semiquinone) and reduced (hydrogen) at the same time, yields the semiquinone (Scheme 1b). This reaction corresponds to a dehydrogenation, and is governed by electron transfers (with a change of global DOx), unlike a deprotonation, which is governed by proton transfers (an acidobasic process, for which the DOx is unchanged) and would produce an alcoholate derivative. Such kind of derivative is expected to have a contribution centred around 533 eV in the O 1s core level spectra,^41^*i.e.* close to the contribution we assigned to alcohol groups (532.8 eV). Our assignment is comforted by the observation of a contribution centred at 533.4 eV in a multilayer HHTP deposit at room temperature on Cu(111) (see ESI, Fig. S12†), the shift (533.4 eV *vs.* 532.8 eV) presumably corresponding to a typical polarisation screening effect.^42^ For this reason we rule out the formation of an alcoholate derivative. For the semiquinone, the global nominal DOx is increased to +3 compared to HHTP, with the specific DOx of C atoms forming double bonds with O atoms increased to +2 (Scheme 1a).

We note that this chemical state of the semiquinone corresponds to an odd number (three) of dehydrogenations. It contrasts with the even number of dehydrogenations per HHTP molecule reported on Ag(111), which was suggested to be stabilised by dominating intermolecular interactions.^28^ On our Cu(111) substrate, strong molecule–substrate bonds presumably counterbalance this trend.

As the temperature is raised above room temperature, starting from 450 K, the O 1s core level spectra show a progressive increase of intensity and decrease of the binding energy of the ketone component (see ESI, Fig. S11†, the data were acquired starting from a multilayer of HHTP molecules and not from a submonolayer as in Fig. 2). While such changes are already observed at room temperature, after annealing at 530 K, the O 1s core level spectrum is left with a single component (see ESI, Table S2†). The binding energy of this component is close, but 0.4 eV lower, to the ketone component in the semiquinone form (*i.e.* before annealing). We conclude that all alcohol functions are dehydrogenated and have the same local DOx, yielding a global nominal DOx of +6, thus corresponding to a quinone (Scheme 1a, bottom line; note that on Fig. 2, bottom panel, we only represent one of the quinone mesomeric forms for reasons that will become clearer later). Here again, the reaction that produces the quinone starting from the semiquinone is an intramolecular redox reaction (Scheme 1c). We would like to highlight that obtaining a quinone, which corresponds to the fully-oxidised form of the starting catechol, is remarkable, and at variance to the only partial oxidation achievable on Ag(111) (ref. 33) or the absence of oxidation on Au(111).^26^ We note that the C 1s core level spectrum we obtained after annealing is also globally shifted to lower binding energy compared to the situation before annealing (see ESI, Table S2†). This is a possible signature of electrons transferred from the substrate towards the molecule, an expected result since the quinone is a strong oxidant and the Cu substrate a large electron reservoir.

### Molecular structure and charge distribution patterns

**Fig. 3:**
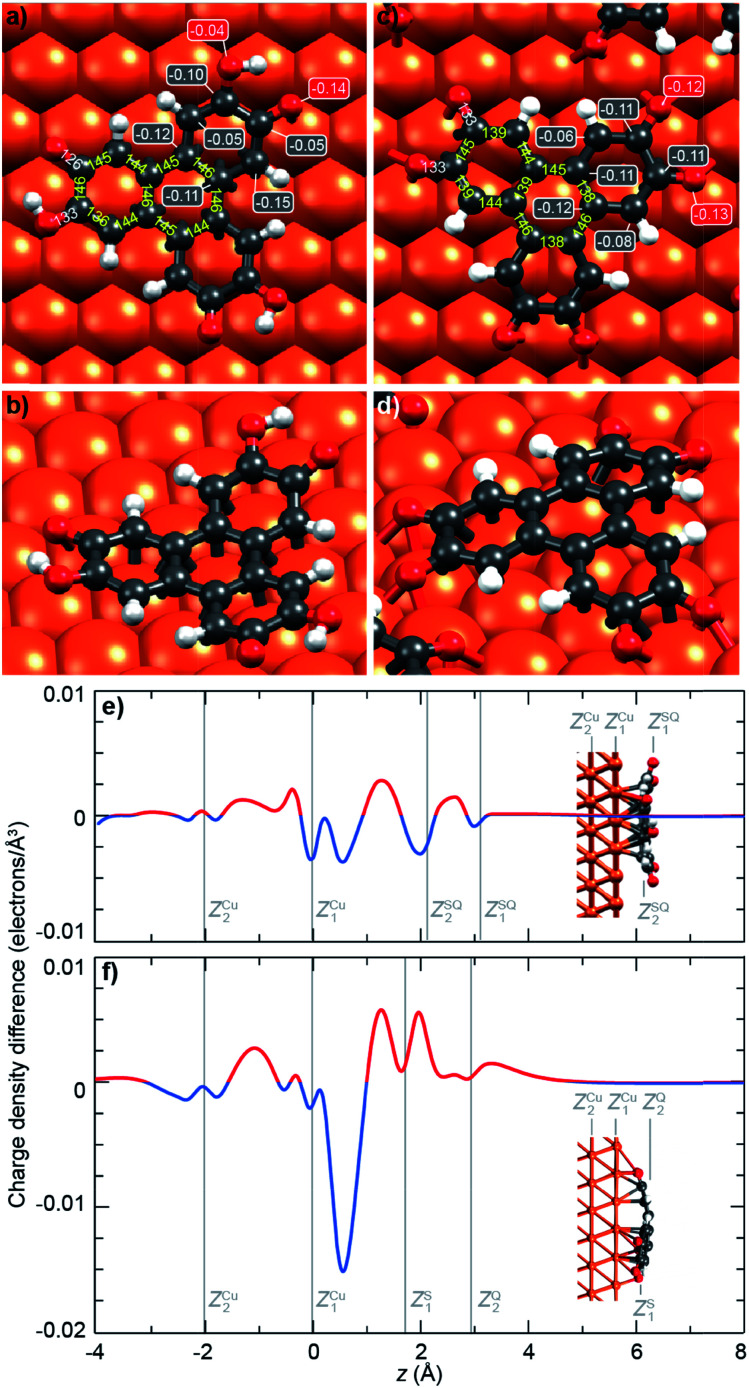
Structure and charge localisation in the molecules. (a, b) Semiquinone (c, d) and quinone forms of the HHTP molecule, as inferred from DFT calculations. (a, c) Top views of the optimised structures, including the most representative bond lengths (yellow positive numbers), and the partial charge held by C atoms bond to O atoms (negative numbers in boxes). (b, d) Perspective views, showing the bowl and dome shapes of the semiquinone and quinone forms. (e, f) Computed charge density difference (accumulation: red; depletion: blue) for (e) the semiquinone and (f) the quinone, projected along the surface normal (*z* direction). Vertical gray lines indicate the second and topmost Cu layers, and the bottom and top part of the molecules.

To get further insights into the intramolecular charge distribution, we now discuss the structure of the molecule as derived from DFT calculations including the substrate. Here, the structural and chemical analysis performed with STM, XPS and NEXAFS provide a crucial input to build a series of relevant starting point structures. Top-views of the optimised structures corresponding to the molecules before and after thermal annealing are displayed in Fig. 3a and c. These figures also include the values of bond lengths.

In Fig. 3a, we observe that, for the semiquinone form (room temperature deposition, without further annealing), all bond lengths are similar to those of the starting gas phase HTTP molecule (fully hydrogenated, see ESI, Fig. S14†), except the larger C–C bonds involving C atoms in the ketone group (not in the alcohol group). This larger bond length is expected since for both molecular semiquinone forms (top of Scheme 1a, middle and right), it is a single C–C bond. The bond lengths in the central ring correspond to an aromatic ring (like in the starting HHTP molecule), suggesting that the observed semiquinone form resembles the SQ1 mesomer sketched in Scheme 1a. The DFT calculations also indicate that the molecule presents a bowl shape on the surface (Fig. 3b and d) due to the bonding to the substrate *via* the central C ring and not *via* the dehydrogenated O atoms. This behaviour is consistent with the radical character that the SQ1 form in Scheme 1a would have in a hypothetical gas phase.

In Fig. 3c, we observe that for the quinone form (obtained after annealing at 530 K), the central C ring features bond lengths close to those of the aromatic ring in the starting gas phase HHTP molecule (fully hydrogenated, see ESI, Fig. S14†) but with a more marked alternation of shorter (138–139 pm) and longer (145–146 pm) C bonds. This alternation is preserved in the three outer rings, in contrast to the lengths observed for the gas phase molecule (two short and four long). Noteworthy, the CO bonds are here larger than in the case of dehydrogenated alcohol function in the semiquinone (Fig. 3a). Such a bond length (133 pm) is closer to the one expected for single C–O bonds than for double C=O bonds.^43^

A similar situation was encountered in much related molecules on Cu(111), dihydroxy-benzoquinone,^30^ pentacenequinone and pentacenetetrone.^39^ At this point, it should be reminded that the ketone signature in the O 1s core level spectrum was found centred at lower binding energy (−0.4 eV) than in the case of the semiquinone. Such a correlation is reminiscent of the one noticed by Heimel *et al.* when studying ketone-containing molecules onto metal surface with which they interact more or less.^39^ Downwards shifts of ketone O 1s core levels appear to be a signature of a bond elongation, which come together with the formation of bonds between O and Cu atoms. In Scheme 1a (bottom line), this trend appears implicitly: the Q1 mesomer of the quinone nicely complies with the DFT-calculated bond lengths. It corresponds to what would be a hexa-O-radical in a hypothetical gas phase. Note that unlike with linear ketones where the unpaired electron on an O atom comes together with an unpaired electron on a neighbour C atom, the peculiar topology of the HHTP molecule delocalises the latter unpaired electron, which together with other unpaired electrons forms double C=C bonds. The Q1 quinone mesomer is expected to be highly reactive at its O atoms, which suggests that the molecule should preferentially bound to the Cu(111) *via* these atoms. This is precisely what DFT predicts, with a molecule exhibiting a dome shape (Fig. 3d). Interestingly, the situation is at variance with the case of the semiquinone, where the bowl-shape is incompatible with strong O–Cu interactions, this time consistent with the shorter bond lengths matching the values expected for C=O bonds, and with a ketone O 1s core level located at higher binding energy. Hence, depending on the degree of oxidation (dehydrogenation), the HHTP molecule comprises ketone groups in the form of either C=O or C–O–Cu bonds. This kind of transformation, which is usually triggered by light excitation,^44^ is here catalysed by the metallic surface.

Beyond structural information, DFT calculations also inform us on the local partial charge held by each individual atom. For the semiquinone form, we observe an excess charge (0.15 electrons) on the C atoms of the peripheral rings that are at ortho position of the ketone group and meta position of the alcohol group (Fig. 3a). This is yet another resemblance with the radical form SQ1 of the molecule shown in Scheme 1a. We also note that, in the alcohol group, there is a stronger electron accumulation in the C atom than in the O atom, while the opposite is true in the ketone group (Fig. 3a). This seems consistent with the trend expected based on simple DOx arguments. For the quinone form, all O atoms have a strong electron accumulation, but this does not occur to the expense of a smaller electron accumulation for their neighbouring C atoms, which also present a strong electron accumulation (Fig. 3b). Thus, these values of the excess electrons should be rationalised in the context of charge transfer with the substrate.

### Electron transfer from the substrate

Given the strong oxidant character of the semiquinone and of the quinone, we expect significant global electron donation from the Cu substrate, which represents an electron reservoir. This is confirmed in Fig. 3e and f, which show the charge density difference for the semiquinone and the quinone projected along the surface normal, as computed by DFT. Positive (negative) values of this quantity indicate regions of charge accumulation (depletion), see more details are given in the ESI, Fig. S7.† We find that the charge transferred from the substrate amounts to 1.2 and 1.9 electrons per molecule for the semiquinone and quinone forms, respectively. These values are large, already exceeding those obtained with the best molecular electron acceptors.^28,45^ In core level spectra, the increased electronic density around the probed atoms, corresponding to the larger electron donation from the substrate (from half-dehydrogenated to fully-dehydrogenated HHTP), is consistent with the global shift to lower binding energies, −0.2 eV for C 1s core level spectra, and −0.4 eV for the ketone contribution to the O 1s core level spectra. In the case of the semiquinone, charge accumulation is found below and on top of the central C ring, which is closer to the surface (see Fig. 3e). This accumulation is compensated by a depletion in the Cu surface, mostly located at the level of the topmost Cu plane and immediately above it. In the case of the quinone, a slight charge accumulation is also predicted in the central C ring (Fig. 3f), but the stronger one is located below and above the O atoms (Fig. 3f), *i.e.* here again where the molecule is closest to the surface. In contrast, there is an important electron depletion slightly above the topmost Cu layer.

### Discussion on the redox behaviour of HHTP adsorbed on a Cu(111) surface

Our results indicate that HHTP can be transformed into a semiquinone (DOx = +3) and hydrogen without any other co-reactant (Fig. 1a–c and ESI, Fig. S3†), already at room temperature upon adsorption on a Cu(111) surface. The semiquinone can then be thermally-converted in a second step into a quinone and hydrogen. These two reactions are instances of intramolecular redox reactions. Upon adsorption on Cu(111), the semiquinone and quinone forms are able to interact with the surface, *via* a substantial surface-to-molecule charge transfer. In this view, Cu(111) behaves as the catalyst of the intramolecular redox reactions. Such a catalytic effect is not found with all metal surfaces, since for example the HHTP molecule is not expected to be dehydrogenated onto Au(111).^26^

The charge transfers apparent in the numerical simulations and XPS data corroborate that the adjustment of the electronic properties of HHTP is the driving force of the adsorption. Charge transfers may alter the energy of the molecular orbitals, hence the interaction between Cu(111) and the molecule, for instance their electronic hybridisation, and in turn chemical transformations. We note that the starting HHTP molecule, being rich in delocalised π electrons, reaching out of the molecular plane, is not liable to experience an attractive interaction with the Cu(111) surface, which is also rich in delocalised electrons. After the three and six surface-assisted dehydrogenation reactions (corresponding to the loss of 3 and 6 electrons respectively), HHTPs are converted into electron-deficient molecules, namely the semiquinone and the quinone.

We suggest that Cu nanoparticles can catalyse the conversion of defensive antioxidants into aggressive oxidants by an alternative pathway to the Fenton and Haber–Weiss reactions. This third pathway, already active at room temperature, does not require a precursor but features a concomitant oxidation and reduction of the molecules, and could explain the cytotoxic behaviour of the metallic (non ionic) nanoparticles by the adjustment of the electronic properties of defensive antioxidant catechols with those of the metal nanoparticles.

## Conclusions

We have resolved the chemical and oxidation state changes of the HHTP molecule experiencing progressive oxidation *via* dehydrogenations on a Cu(111) surface. Unlike metal cations,^46–49^ the Cu substrate is unable to oxidise the starting catechol. On Cu(111), we find that the latter exhibits an ambivalent redox role, behaving as both an oxidising (C atoms) and a reducing (H atoms in the starting alcohol functions) agent. In other words, it experiences two on-surface intramolecular redox reactions (*i.e.* surface-assisted dehydrogenation) yielding hydrogen radicals, a semiquinone (half alcohol functions oxidised) and eventually a quinone (all six alcohol functions oxidised). To our knowledge, these forms could not be obtained on other metal surfaces such as Au(111) and Ag(111).^26,30,33^ Once oxidised, the molecules readily accept electrons from the substrate, in large amounts (about 1 and 2 per molecule respectively), consistent with their strong oxidant character. Temperature control is the key to achieve the highest degree of oxidation of the molecule. This is a rather unusual observation, suggesting that the second redox reaction is kinetically limited at room temperature. Our findings suggest that metal surfaces, such as those exhibited by metal nanoparticles, may promote a (self-)conversion of antioxidant molecules such as catechols into highly reactive oxygen species, thanks to intramolecular redox reactions, driven by the adjustment of the electronic properties of the molecule/Cu nanoparticle system. The semiquinone form of HHTP is already a strong oxidant, forming spontaneously at room temperature. The quinone is an even stronger oxidant, which, according to our analysis performed in model conditions, may form already at 450 K. Whether the formation of the quinone could occur even closer to body temperature, for instance in contact with other kinds of surfaces (other surface terminations, other kinds of metal) or in the presence of other species in the surrounding, is an open question. Our work suggests a route to the promotion of oxidative stress by nanoparticles, this time involving the ubiquitous catechol family. Together with their deleterious effect on extra- and intra-vasation of cancer cells,^50^ this extends the panorama of potential dangers of nanoparticles with respect to the development of cancers and other degenerative diseases. Beyond these potential health issues, it is also worth noting that, with their specific charge distribution making them suitable building-blocks for electrically conductive crystalline molecular frameworks,^47–49,51^ the molecules may possess long-sought spin distributions also making them candidate building-blocks for ferromagnetic molecular frameworks.^46^ These magnetic properties are related to their radical character in the gas phase,^3,52^ and they might be scrutinised in the future with the help of high resolution spatially resolved surface science spectroscopy techniques.^53^

## Experimental and numerical methods

### Materials

2,3,6,7,10,11-Hexahydroxytriphenylene (HHTP, 95% purity) was purchased from TCI-Europe. The compound was purified at the laboratory by repeated washing in organic solvents until a purity of >99% was reached, as checked with NMR. The substrate was a copper single crystal (99.9999% purity) cut with a (111) surface, provided by SPL. To prepare a clean Cu(111) surface we used Ar ion bombardment at 800 eV followed by annealing to 620–670 K (measured with an infrared pyrometer, emissivity value set to 10%), all under UHV. The surface cleanliness and the size of the atomically flat terraces were checked with STM, low-energy electron diffraction (LEED), RHEED, and XPS in the different UHV chambers that we used.

### Ultrahigh vacuum on-surface deposition

To deposit the HHTP molecules we used a commercial Kentax evaporator and home-made evaporators made of resistively heated tantalum pockets. After extensive degassing of the molecular evaporator loaded with molecules in a separate vacuum chamber for a day, we inserted the evaporators in the UHV chambers (Grenoble, Madrid, Trieste). We first roughly adjusted the evaporator temperature to detect a modification of the diffraction pattern of clean Cu(111) and whenever possible with a quartz microbalance. To calibrate the deposition rate, we next finely adjusted the evaporator temperature to obtain a sub-monolayer deposit on Cu(111) within a given time, as observed with STM. Molecule deposition on the sample was performed at room temperature and the sample was analysed before and after annealing to 530 K for 30 min (temperature measured with pyrometers and thermocouples).

### Scanning tunneling microscopy

We used chemically-etched W tips and mechanically-prepared PtIr tips. Two STM systems were used, both connected under UHV to a preparation chamber (UHV surface preparation and molecular deposition): a flow-cryostat instrument from SIGMA Surface Science operated at 79 K (STREAM), and a room temperature Omicron STM 1 instrument. The images were processed with the Gwyddion software, and corrected from thermal drift effects by analysing images of the same sample region acquired subsequently with opposite scanning speeds in the slow-raster direction. Electron diffraction (RHEED, LEED) were used to infer the main crystallographic directions of the Cu(111) surface.

### X-ray photoemission spectroscopy and near-edge X-ray absorption spectroscopy

XPS and NEXAFS experiments were performed at the ALOISA beamline of the Elettra synchrotron facility.^54^ C 1s, O 1s and Cu 3p core level spectra were recorded. We used 600 eV and 400 eV photon energies. For NEXAFS measurements, absolute energy calibration was done by systematic acquisition of the drain current of the last (Au-coated) refocusing mirror. The XPS spectra were normalised by this current. The binding energy scale was calibrated to the Cu reference core level at 75.1 eV. Polarisation-dependent NEXAFS measurements were performed at the O and C K-shell ionisation thresholds in partial electron yield by means of a full aperture detector (channeltron) in front of the sample with an electrostatic high-pass filter set to −200 V to reject secondary electrons. NEXAFS were acquired in transverse electric (*s* polarisation) and transverse magnetic (quasi *p* polarisation) by rotating the sample around the photon beam axis while keeping the angle grazing at 6°.

### Density functional theory calculations

The structure and electronic charge distribution of the molecules on Cu(111) were optimised for the semiquinone and quinone forms of HHTP using the QUANTUM ESPRESSO plane-wave DFT code.^55^ The Cu(111) substrate was modelled using four in-plane-periodic layers, the atomic positions in the two bottom ones being fixed during the structural relaxation process. The calculations take into account an empirical efficient van der Waals *R*-6 correction (DFT+D2 method^56^). Electronic exchange and correlation effects were accounted for using the generalised gradient approximation PBE functional.^57^ Ultrasoft pseudo potentials were used to model the ion–electron interaction within the H, C, O and Cu atoms.^58,59^ The Brillouin zones were sampled using optimal Monkhorst–Pack grids.^60^ The one-electron wave-functions were expanded in a basis of plane waves with 450 eV cutoff for the kinetic energy. Atomic relaxations were performed until the maximum force acting on any atom was below 0.05 eV Å^−1^. Simulations of the STM images were performed within the Keldysh–Green function formalism^61^ as implemented in the localised-basis set code FIREBALL,^62^ where both sample and tip contributions are explicitly accounted for.

## Author contributions

A. C. G. H. and C. S. performed the STM measurements. F. C. purified the molecules. J. A., L. F., A. V., A. C. and J. A. M. G. performed the XPS and NEXAFS measurements. A. C. G. H. and C. S. performed the XPS and NEXAFS analysis. J. I. M. performed the DFT calculations and STM image simulations. E. M., S. L., V. G., P. D. and J. C. provided assistance during the UHV experiments. A. C. G. H., C. S., F. C., J. A. M. G., J. C. wrote the manuscript with inputs from all authors. J. C. coordinated the writing of the manuscript.

## Conflicts of interest

There are no conflicts to declare.

## Supplementary Material

SC-012-D0SC04883F-s001
