# Supplementary material for: Copper-assisted oxidation of catechols into quinone derivatives
Source: Chem Sci. 2020 Dec 21;12(6):2257–67. doi: 10.1039/d0sc04883f (PMC8179264; doi:10.1039/d0sc04883f)
Supplement: SC-012-D0SC04883F-s001 [file SC-012-D0SC04883F-s001.pdf]

Electronic Supplementary Material (ESI) for Chemical Science.
This journal is © The Royal Society of Chemistry 2020

Electronic Supplementary Information

# Copper-assisted oxidation of catechols into quinone derivatives

Ana Cristina Gómez-Herrero,[a] Carlos Sánchez-Sánchez,[b] Frédéric Chérioux,[c] Jose Ignacio Martínez,[b] José Abad,[d] Luca Floreano,[e] Alberto Verdini,[e] Albano Cossaro,[e] Estelle Mazaleyrat,[f] Valérie Guisset,[a] Philippe David,[a] Simone Lisi,[a] José Angel Martín Gago[b] and Johann Coraux[a]

a. Univ. Grenoble Alpes, CNRS, Grenoble INP, Institut NEEL, 38000 Grenoble, France.

b. Materials Science Factory, Instituto de Ciencia de Materiales de Madrid-CSIC, C/Sor Juana Inés de la Cruz 3, Madrid 28049, Spain.

c. Univ. Bourgogne Franche-Comté, FEMTO-ST, CNRS, UFC, 15B avenue des Montboucons, F-25030 Besançon Cedex, France.

d. Departamento de Física Aplicada, Universidad Politécnica de Cartagena, Calle Doctor Fleming, s/n,30202 Cartagena, Spain.

e. Laboratorio TASC, CNR-IOM, Basovizza SS-14, Km 163.5, 34149 Trieste, Italy.

f. Univ. Grenoble Alpes, CEA, IRIG/DEPHY/PHELIQS, 38000 Grenoble, France.

**Table of contents**


- Known redox reactions producing reactive oxygen species in presence of copper ions
- Monoradical form of the seminquinone
- DFT simulations of the semiquinone and quinone forms of HHTP on Cu(111) and STM image simulation
- Second supramolecular phase observed after room temperature deposition on Cu(111)
- Procedure for the analysis of the XPS data
- NEXAFS spectra after room temperature deposition of HHTP molecules
- Structure of the starting HHTP molecule in the gas phase
- References

## Known redox reactions producing reactive oxygen species in presence of copper ions

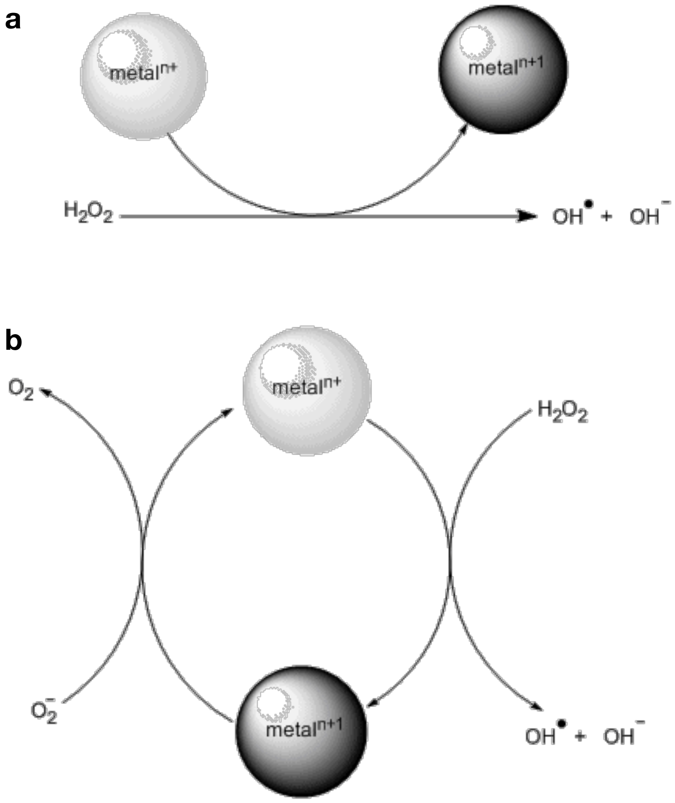


**Fig. S1** (a) Fenton-type reaction. The reduction of hydrogen peroxide into highly reactive hydroxyl radical and hydroxide anion induces the oxidation of metal cations (mainly $Cu^{+}$ or $Fe^{2+}$) into corre-sponding metal cations bearing an additional positive charge. (b) Haber-Weiss type reaction. The hydrogen peroxide is also reduced into highly reactive hydroxyl radical and hydroxide anion with oxidation of metal cations bearing an additional positive charge and production of $O_2$.

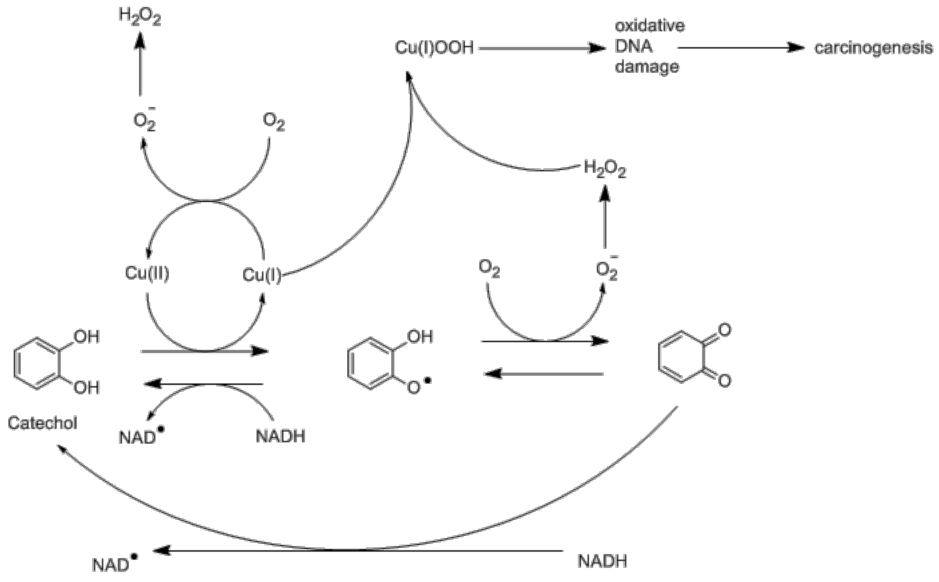


**Fig. S2** Hypothetic mechanism of oxidative DNA damage induced by catechol and $Cu^{2+}$.

The Fenton or Haber-Weiss reactions consist of redox processes involving metal cations, as illustrated in Fig. S1 in the case of hydrogen peroxide and Cu cations.

The mechanism of oxidative DNA damage induced by catechols has been investigated in the literature (see, *e.g.* Ref. 1). In this mechanism, a catechol is oxidised into a semiquinone and a quinone with the generation of $H_2O_2$. In this chemical pathway, $Cu^{2+}$ are reduced into $Cu^{+}$, the latter contributing to the oxidative damage of DNA (Fig. S2). This mechanism is very different from Haber-Weiss or Fenton reactions, because it requires the reduction of $Cu^{2+}$ into $Cu^{+}$ while Haber-Weiss or Fenton are based on the oxidation of $Cu^{+}$ into $Cu^{2+}$. This mechanism is *a priori* impossible in the case of Cu nanoparticles because copper atoms are in the Cu(0) form, which cannot be reduced.

## Monoradical form of the semiquinone

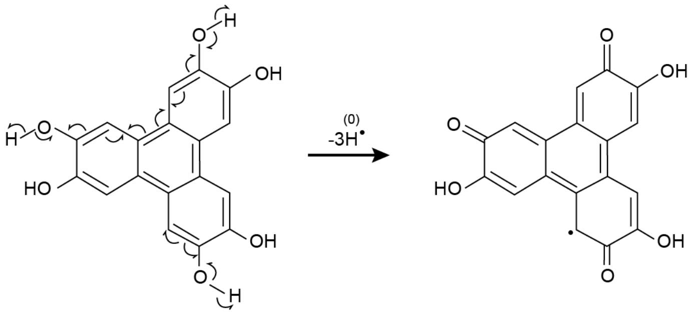


**Fig. S3** Alternative path to the oxidation of the HHTP molecule into a semiquinone. For simplicity, we do not show the other possible forms like we did in Fig. 1 of the main text.

Contrary to the case sketched in Fig. 1 of the main text, the dehydrogenation of the HHTP molecule may occur in a way that breaks the three fold symmetry of the molecule, but still altering all three starting catechol groups. Such a situation is depicted in Fig. S3. In such a case, the charge reorganisation within the semiquinone gives it a monoradical character, and not a triradical character as in the case of Fig. 1 of the main text.

One may wish to seek for STM evidences of one form or the other (Fig. 1 of the main text *versus* Fig. S3), however, our STM image simulations of the semiquinone molecules (see next section) reveal that the ketone and alcohol groups should be indiscernible. We hence cannot tell which form (monoradical or triradical) is actually formed on Cu(111) based on the experimental techniques that we used.

## DFT calculations of the semiquinone and quinone forms of HHTP on Cu(111) and STM image simulation

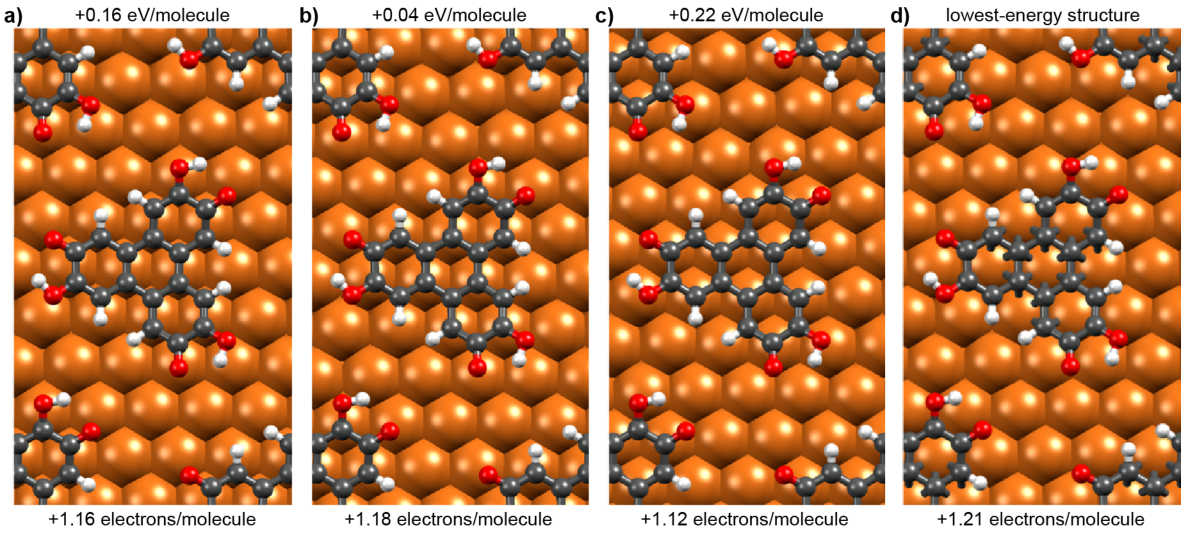


**Fig. S4** Top-view structure of half-dehydrogenated (semiquinone) HHTP on Cu(111), in a (5×5) unit cell, attached to the surface at different binding site, with the central C ring lying on (a) bridge, (b) hollow *fcc*, (c) hollow *hcp* and (d) *top* sites, as optimised with DFT calculations. Total energy differences with respect to the most stable configuration (d) and charge transferred from the Cu(111) surface to the molecules are indicated respectively on top and under each panel.

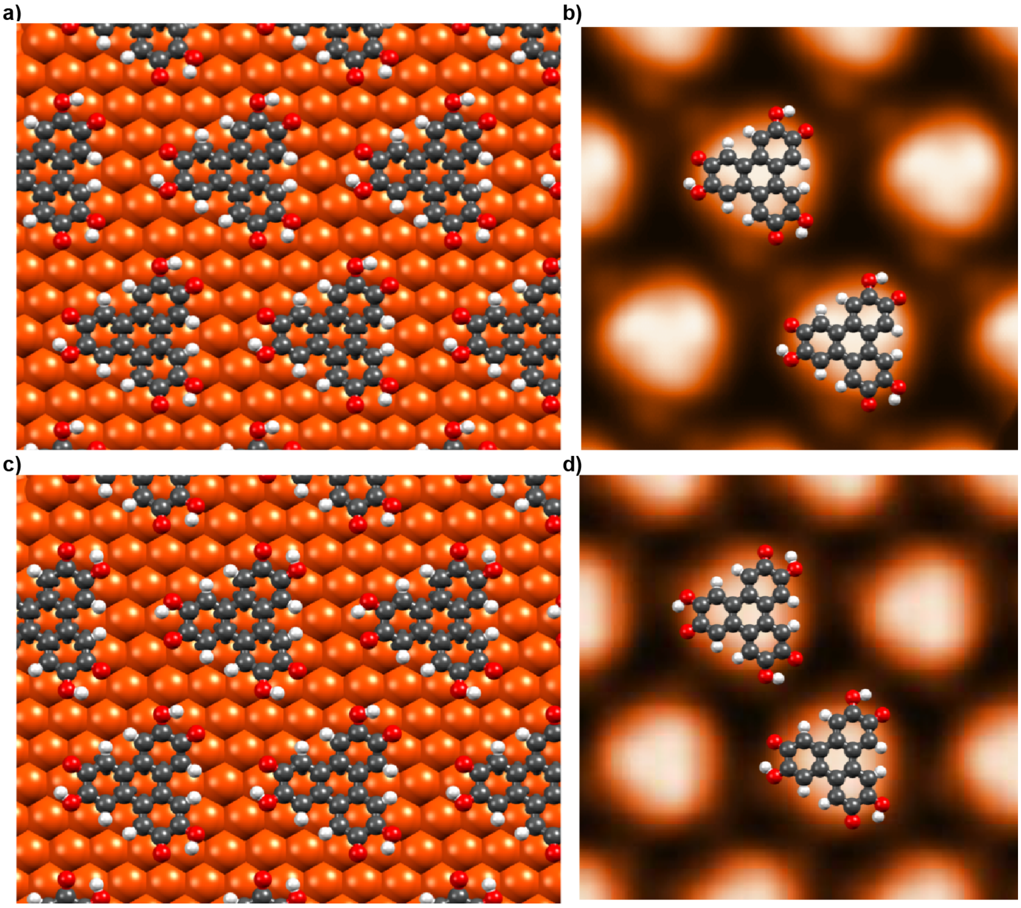


**Fig. S5** Top-view structure of half-dehydrogenated (semiquinone) HHTP on Cu(111), in a (5×5) (a) and in a (5×10) (c) unit cell, as optimised with DFT calculations. In the latter case, every second molecule is a different enantiomer, with a different set of triplet of O-H bonds. (b,c) Corresponding simulated STM images (0.1 nA, -1 V).

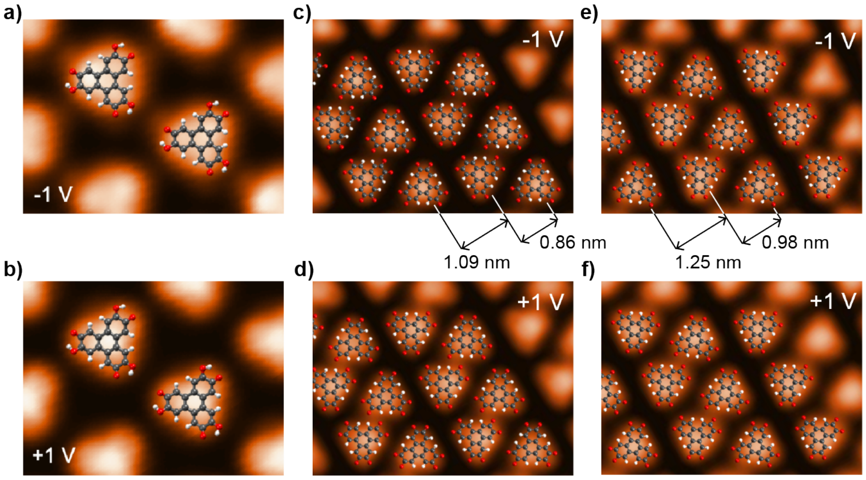


**Fig. S6** STM Image simulation based on the DFT-optimised structure of the semiquinone (a,b) and quinone (c-f) forms of HHTP on Cu(111) (c,d: (5×9), e,f: (5×10)), for a tunnel bias of -1 V (a,c,e) and +1 V (b,d,f).

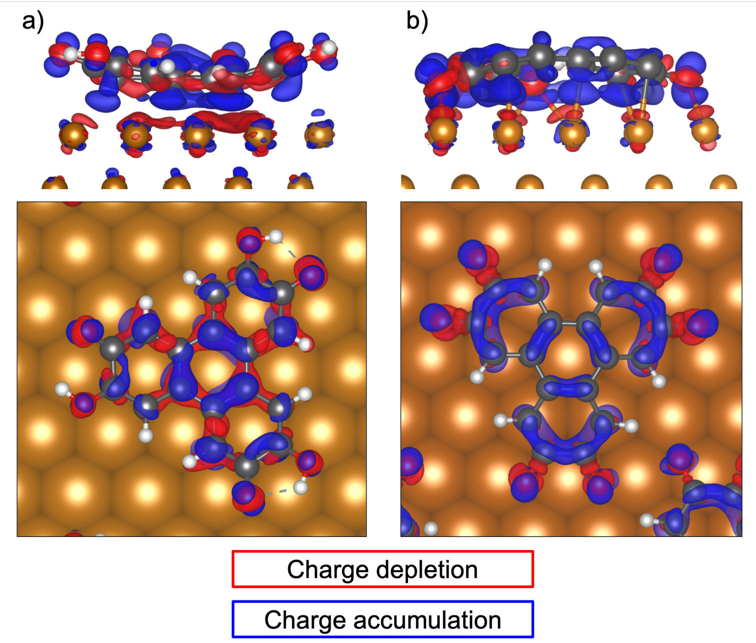


**Fig. S7** Top- and side- views of the three-dimensional isosurfaces for the semiquinone (a) and quinone (b) forms of HHTP optimised by the DFT calculations, corresponding to the charge density difference, $\Delta\rho(\vec{r}) = \rho_{\text{total}}(\vec{r}) - [\rho_{\text{molecule}}(\vec{r}) + \rho_{\text{surface}}(\vec{r})]$, in both interfaces (blue, +0.005 electrons/Å$^3$: charge accumulation; red, -0.005 electrons/Å$^3$: charge depletion).

We first tested several possible binding sites for the semiquinone form of HHTP on Cu(111) in a (5×5) Cu(111) unit cell. In these different phases, the center of the central C ring of the molecule lies:

a. on *bridge* sites of Cu(111) (Fig. S4a),
b. on hollow *fcc* sites of Cu(111) (Fig. S4b),
c. on hollow *hcp* sites of Cu(111) (Fig. S4c),
d. directly atop a Cu atom (Fig. S4d).

The difference in total energy of these configurations with respect to the most stable configuration (Fig. S4d) is +0.16 eV/molecule (Fig. S4a), +0.04 eV/molecule (Fig. S4b) and +0.22/molecule (Fig. S4c), respectively.

Symmetric half dehydrogenation of HHTP can produce two enantiomers of the semiquinone on Cu(111), that differ only by the location of O-H bonds. We optimised the structure of two supramolecular networks, one with a single kind of enantiomer (Fig. S5a, (5×5) Cu(111) unit cells), and one with a racemic (5×10) phase (Fig. S5b), in both cases corresponding to the networks observed in STM (main text Fig. 2). The latter supramolecular network has higher energy, by about 110 meV/molecule.

With the outcome of the DFT simulation as an input, we computed constant-tunneling-current (0.1 nA) STM images with bias voltage of -1.00 V, 0.05 V, and +1.00 V. For that purpose, we used a Keldysh-Green function formalism with the first-principles tight-binding Hamiltonian obtained from the FIREBALL code.[2-4] We took into account the electronic properties of both the tip and the sample. To simulate the STM tungsten tip, we considered five protruding atoms (one at the apex) attached to an extended W(100) crystal. We used this procedure to simulate STM images of the semiquinone form in the (5×5) superstructure on Cu(111) (Fig. S6a) and of the quinone form in the (5×9) superstructure (Fig. S6c) and (5×10) superstructure (Fig. S6e) on Cu(111). We observe no atomic detail but rather molecular orbitals, as expected. Strikingly, the appearance of the molecules in STM images is similar for the semiquinone and the quinone, the structure of which are however different with one being bowl-shaped (semiquinone) and the other dome-shaped (quinone). We also do not observe a clear difference in the appearance of alcohol and ketone groups. Besides, we observe no clear difference between the two different kinds of quinone mol-

ecules within the (5×9) or (5×10) unit cells, consistent with the STM observations and with the fact that they both bind on the same kind of site on Cu(111). STM image simulations with different binding configurations reveal, in contrast, distinct aspects. This altogether indicates that no striking difference should be seen between the molecules in the two forms in the experimental STM images.

We also performed simulations of the STM images for the two enantiomers of the semiquinone forms, and find no obvious difference (Fig. S5).

Finally, the DFT calculations provide us with the charge distribution in the molecules. In the case of the semiquinone form of HHTP, most of the charge accumulates in the basal planes of the central C-ring, which is closer to the surface (Fig. S7a). For the quinone form, the depletion of charge in the topmost Cu layer is more pronounced, consistent with a stronger charge transfer from the substrate, and it distributes mainly in the unsaturated molecular O atoms contacting with the metal surface and in the outmost C—C bonds linked to the oxygen atoms in the external C-rings (Fig. S7b).

## Second supramolecular phase observed after room temperature deposition on Cu(111)

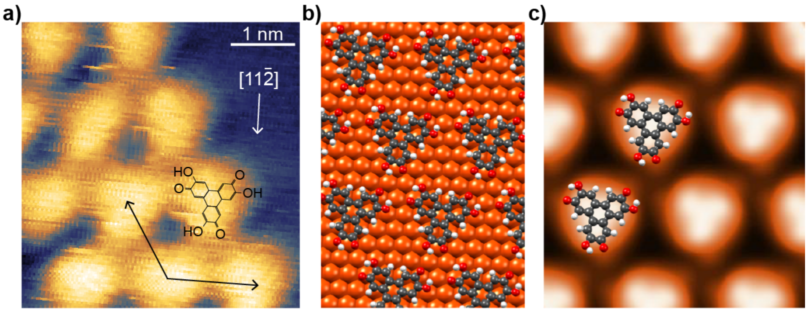


**Fig. S8** (a) STM Image of the $\beta$-type supramolecular (5×5) phase of half-dehydrogenated HHTP molecules (semiquinone form) on Cu(111), observed after room temperature deposition on Cu(111). (b) Corresponding top view of the structure optimised with DFT calculations. (c) Simulated STM image based on the DFT-optimized structure of (b) in constant-tunnel-current regime (0.1 nA), for a -1 V tunnel bias.

Figure S8 shows a STM image of the minority supramolecular phase coexisting with the one discussed in the main text, after room temperature deposition of the HHTP molecules and without further annealing. The $[11\bar{2}]$ direction of Cu(111) almost matches the orientation of one median of the triangular molecular units.

## Electron diffraction data for the fully dehydrogenated molecules

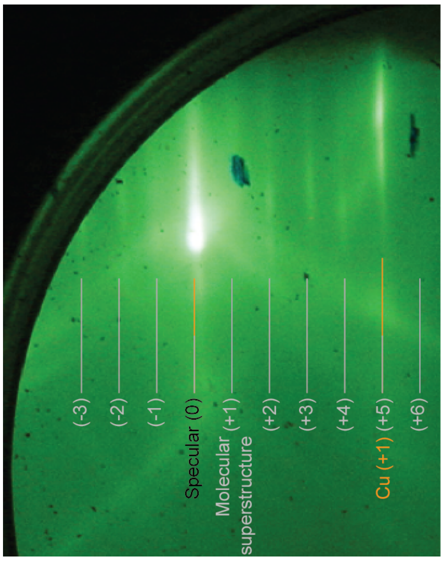


**Fig. S9** RHEED pattern acquired along the $\langle 11\bar{2} \rangle$ azimuth after the deposition of a sub-mono-layer coverage of HHTP molecules on Cu(111) at room temperature and a subsequent annealing at 530 K.

Figure S9 shows a RHEED pattern acquired for fully-dehydrogenated HHTP molecules on Cu(111). In addition to the Cu-related streaks, which were present before molecule deposition, a pattern of streaks with a ×1/5 spacing compared to the spacing between Cu streaks is observed. The streaks are labeled according to their diffraction order with respect to the molecular lattice (gray numbers in parenthesis) or with respect to the copper lattice (orange numbers in parenthesis).

## Procedure for the analysis of the XPS data

| C 1*s* (four-component fit) | | | | |
|---|---|---|---|---|
| Component positions (eV) | 285.8 | 285.6 | 284.6 | 284.2 |
| Gaussian FWHM (eV) | 0.44 | 0.44 | 0.40 | 0.54 |
| Area under component | 0.3 | 0.3 | 0.9 | 1.0 |
| C 1*s* (three-component fit) | | | | |
| Component postions (eV) | | 285.7 | 284.7 | 284.2 |
| Gaussian FWHM (eV) | | 0.65 | 0.47 | 0.6 |
| Area under component | | 0.9 | 1.1 | 1.0 |
| O 1*s* | | | | |
| Component postions (eV) | | 532.8 | 530.8 | |
| Gaussian FWHM (eV) | | 1.2 | 1.4 | |
| Area under component | | 1.0 | 1.0 | |

**Table S1** Refined fit parameters used to produce the best fit to the C 1s and O 1s core level spectra measured after room temperature deposition of the molecules. The area under the different components is also given.

| C 1*s* | | | |
|---|---|---|---|
| Component postions (eV) | 285.7 | 284.4 | 284.0 |
| Gaussian FWHM (eV) | 0.5 | 0.4 | 0.5 |
| Area under component | 0.8 | 1.4 | 1.0 |
| O 1*s* | | | |
| Component postions (eV) | 530.4 | | |
| Gaussian FWHM (eV) | 1.0 | | |
| Area under component | 1.8 | | |

**Table S2** Refined fit parameters used to produce the best fit to the C 1s and O 1s core level spectra measured after room temperature deposition of the molecules and subsequent 530 K annealing. The area under the different components is also given.

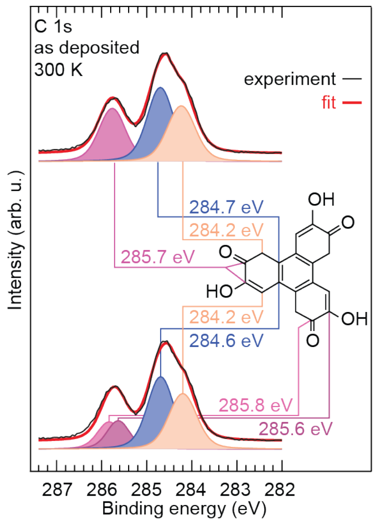


**Fig. S10** C 1*s* core level spectra acquired for a sub-monolayer deposit of HHTP molecules on Cu(111) at room temperature. The experimental (black curves) data are fit (red curves) with three (top, same as in Fig. 2 of the main text) and four (bottom) components.

The spectra presented in Fig. 2 of the main text have been subtracted with a Shirley-type background. Multi-component fits of the experimental data were obtained by Voigt functions, assuming a fixed full width at half maximum (FWHM) of the Lorentzian part of 0.2 eV and 0.3 eV for the C 1s and O 1s core levels respectively. The refined values of the FWHM of the Gaussian part of the Voigt functions were found in the range of 0.5-1.2 eV. These values and the peak centre positions are summarised in the two Tables S1 and S2 in the case of the molecules deposited at room temperature before and after annealing at 530 K.

The relative areas under the different components of the peaks are consistent with the relative number of C or O atoms associated to each component.

For the C 1s core level spectra corresponding to the room-temperature deposit (before annealing), we performed two kinds of fits (Fig. S10), with one or two components accounting for the peak observed at higher binding energy. By considering two components (altogether four-component fit), we assume that two chemically-inequivalent C atoms are bound to O atoms, those involved in C-O-H and in C=O bonds. The fit yields equal weight for the two components, which is consistent with a half-dehydrogenation of the molecules (Table S1). The binding energy of the other components, corresponding to C atoms not bound with O atoms, are hardly affected by the kind of fit (with altogether three or four components), see Table S1.

XPS data were fit with a code written for Igor (developed by Francesco Bruno, ALOISA beamline, Laboratorio TASC, CNR-IOM).

## Additional O 1s core level spectra

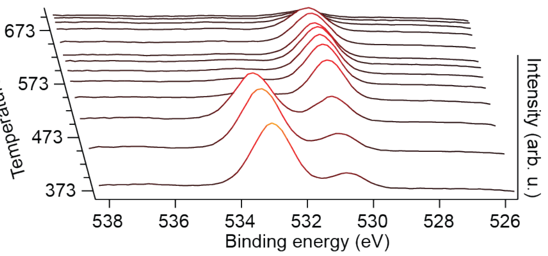


**Fig. S11** Series of O 1s core level spectra measured as a function of the temperature, after a multilayer deposit of HHTP molecules has been performed at room temperature.

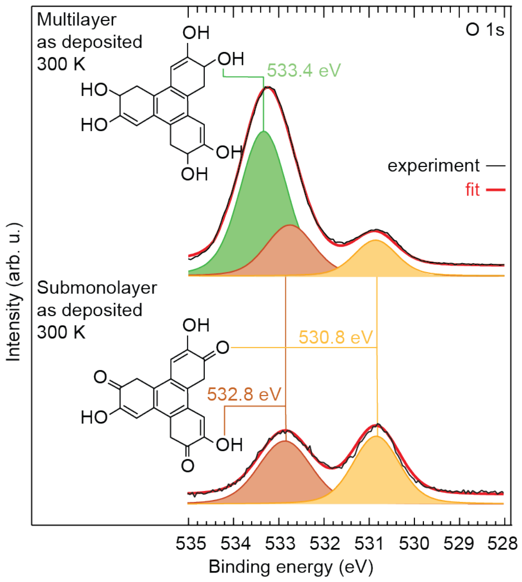


**Fig. S12** O 1*s* core level spectra acquired for a multilayer (top) and a sub-monolayer (bottom) deposit of HHTP molecules at room temperature (bottom, same data as in Fig. 2 of the main text). The experimental data (black curves) are fit (red curve) with several components, each corresponding to chemically inequivalent O atoms.

After having deposited a multilayer of HHTP molecules onto Cu(111) at room temperature, we increased the sample temperature step-by-step, and acquired O 1s core level spectra at each step (Fig. S11). Please note that the spectrum measured here at 373 K has a very intense high binding energy component, much stronger than the one observed in Fig. 2 of the main text for a room temperature deposit. This is not a contradiction, since the spectrum measured here (Fig. S11) concerns a multilayer deposit of HHTP molecules, where most molecules are fully hydrogenated (see analysis of Fig. S12 below), while Fig. 2 concerns a sub-monolayer deposit of HHTP molecules, where all molecules are already half-dehydrogenated at room temperature.

In Fig. S11, we observe a progressive decrease of the intensity of the high binding energy component, starting from 450 K, until it vanishes (530 K). At least a significant fraction of the decrease of the intensity *versus* temperature in this binding energy range arrises from the desorption of molecules not directly in contact with the substrate. More directly informative concerning the chemical modification of the molecules in contact with Cu(111), the lower binding energy peak increases in intensity with temperature (starting from 450 K) and shifts to lower binding energy. Eventually, at the highest temperatures, the absence of signal points towards a desorption or decomposition of the molecules that were in contact with Cu(111).

In Fig. S12, we compare the O 1s core level spectra for HHTP molecules deposited on Cu(111) at room temperature, either in the form of a multilayer or in the form of a sub-monolayer. The multilayer consists of one monolayer in direct contact with Cu(111), and of several layers with no contact to it. For the former, we assume the same components (C-**O**-H, C=**O**) as those found in the sub-monolayer deposit. The C-**O**-H function for the non-dehydrogenated molecules (within the multilayer) has a contribution centred at slightly higher binding energy (533.4 *vs* 532.8 eV in the monolayer).

## NEXAFS spectra after room temperature deposition of HHTP molecules

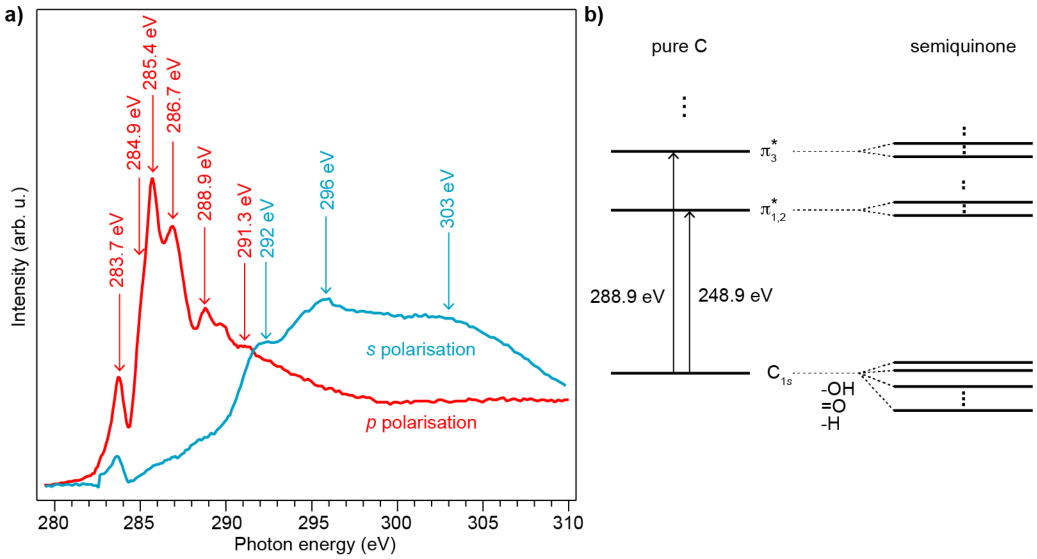


**Fig. S13** (a) C 1*s* K-edge NEXAFS spectra acquired with a *p* polarisation (electric field perpendicular to the surface) and *s* polarisation (electric field parallel to the surface), after the room temperature deposition of HHTP molecules. (b) Schematic diagram of the electronic states of the semiquinone form of the HHTP molecule (the form of the molecule after room temperature deposition on Cu(111)), compared to the case of pure carbon, adapted from Ref. 2.

Transitions between occupied and unoccupied molecular orbitals have characteristic signatures in NEXAFS. Figure S13a shows two spectra measured at the K edge of C, with the polarisation of the electric field parallel (*s* polarisation) and perpendicular (*p* polarisation) to the surface. Several peaks/features are observed.

A first comparison to previously published measurements on simpler related compounds (benzene, phenols and quinones[5-7]) allows assigning part of these peaks/features. The shoulder at 284.9 eV and the peak at 288.9 eV observed with the *p* polarisation are also found in benzene, where they have been ascribed to transitions from the C 1*s* core level to the first, second and third excited $\pi^*$ states (Fig. S13b). For this polarisation, five other peaks, at 283.7 eV, 285.4 eV, 286.7 eV, 289.8 eV and 291.3 eV, are observed that are not found for benzene.

We suggest that the four latter correspond to chemically inequivalent C atoms (Fig. S13b): while in benzene all C atoms are equivalent, for the semiquinone form of HHTP (and the HHTP molecule itself, actually) there are different C environments depending on the bonding atoms (-C, -OH, =O or H). In addition, as discussed in the main text, the interaction with the Cu(111) substrate does not involve all C atoms in the same way.

The former peak (283.7 eV) presumably relates to the molecular charge distribution, being reminiscent of observations in p-benzoquinone, which were interpreted as a signature of a transition to a low-energy molecular C=C-C-O hybrid $\pi^*$ orbital.[5]

Measurements with *s* polarisation reveal broad features, centred at 292 eV, 296 eV and 303 eV, that are characteristic of electronic transitions to $\sigma^*$ states,[8] which are located in the plane of the rings and are usually used to determine the orientation of the molecules. The high intensity of these features, together with the non-vanishing $\pi$ transitions, are consistent with the bowl-shape molecular geometry, with the center of the molecules being parallel to the surface.

In any case, it is noteworthy that we observe a strong anisotropy in the spectra when changing light polarisation, a clear signature of an almost“planar” bonding on the surface.[8,9]

## Structure of the starting HHTP molecule in the gas phase

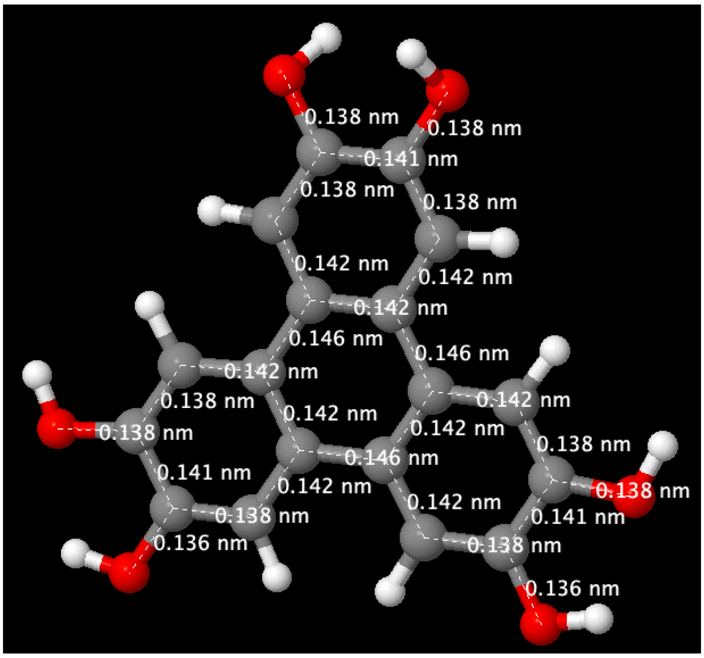


**Fig. S14** DFT-optimised structure of the HHTP molecule in the gas phase, together with the C-C and C-O bond lengths.

Figure S14 shows the DFT-optimised structure of the HHTP molecule in the absence of a substrate (gas phase). The C-C and C-O bond lengths are indicated on the figure. In the central ring, the bond lengths are similar and globally close to the 140 pm value of benzene, pointing towards an aromatic character of the central ring. This also seems to be the case in the peripheral rings, although the bond length dispersion is slightly larger there.

## References


1. S. Oikawa, I. Hirosawa, K. Hirakawa and S. Kawanishi, Site specifity and mechanism of oxidative DNA damage induced by carcinogenic catechol. *Carnogenesis* 2001, **22**, 1239-1245.
2. J. Francis and A. Hitchcock, Inner-shell spectroscopy of p-benoquinone, hydroquinone, and phenol: distinguishing quinoas and benzoin structures. *J. Phys. Chem.* 1992*,* **96**, 6598-6610.
3. J. P. Lewis, P. Jelínek, J. Ortega, A. A. Demkov, D. G. Trabada, B. Haycock, H. Wang, G. Adams, J. K. Tomfohr, E. Abad, H. Wang and D. A. Drabold, Advances and applications in the FIREBALL ab initio tight-binding molecular-dynamics formalism, *Phys. Status Solidi B* 2011, **248**, 1989-2007.
4. J. M. Blanco, C. González, P. Jelínek, J. Ortega, F. Flores and R. Pérez, First-principles simulations of STM images: from tunneling to the contact regime, *Phys. Rev B* 2004, **70**, 085405.
5. J. Solomon and R. Madix, Orientation and absolute coverage of benzene, aniline, and phonon on Ag(110) determined by NEXAFS and XPS. *Surf. Sci.* 1992, **255**, 12-30.
6. D. Solomon, J. Lehmann, J. Kinyangi, B. Liang, K. Heymann, L. D. K. Hanley, S. Wirick and C. Jacobsen, Carbon (1s) NEXAFS spectroscopy of biogeochemically relevant reference organic compounds. *SSSAJ* 2009, **73**, 1817-1830.
7. D. Käfer and G. Witte, Evolution of pentacene films on Ag(111): growth beyond the first monolayer. *Chem. Phys. Lett.* 2007, **442**, 376-383.
8. N. Ruiz del Árbol, I. Palacio, G. Otero-Irurueta, J. I. Martínez, P. L. de Andrés, O. Stetsovych, M. Moro-Lagares, P. Mutombo, M. Svec, P. Jelínek, A. Cossaro, L. Floreano, G. J. Ellis, M. F. López and J. A. Martín-Gago, On-surface bottom-up synthesis of azine derivatives displaying strong acceptor behavior, *Angew. Chem. Int. Ed.* 2018, **130**, 8718-8722.
9. J. Stöhr and D. Outka, Determination of molecular orientations on surfaces from the angular dependence of near-edge X-ray absorption fine-structure spectra. *Phys. Rev. B* 1987, **36**, 7891-7830.
